# Organ-on-a-Chip: A Roadmap for Translational Research in Human and Veterinary Medicine

**DOI:** 10.3390/ijms262110753

**Published:** 2025-11-05

**Authors:** Surina Surina, Aleksandra Chmielewska, Barbara Pratscher, Patricia Freund, Alexandro Rodríguez-Rojas, Iwan Anton Burgener

**Affiliations:** Clinical Department for Small Animals and Horses, Division of Small Animal Internal Medicine, Univesity of Veterinary Medicine, 1210 Vienna, Austria; surina.surina@vetmeduni.ac.at (S.S.); aleksandra.chmielewska@vetmeduni.ac.at (A.C.); barbara.pratscher@vetmeduni.ac.at (B.P.); patricia.freund@vetmeduni.ac.at (P.F.)

**Keywords:** organoids, organ-on-chip, microfluidics, translational medicine, veterinary models

## Abstract

In this review we offer a guide to organ-on-chip (OoC) technologies, covering the full experimental pipeline, from organoid derivation and culture, through microfluidic device fabrication and design strategies, to perfusion systems and data acquisition with AI-assisted analysis. At each stage, we highlight both the advantages and limitations, providing a balanced perspective that aids experimental planning and decision-making. By integrating insights from stem cell biology, bioengineering, and computational analytics, this review presents a compilation of the state of the art of OoC research. It emphasizes practical considerations for experimental design, reproducibility, and functional readouts while also exploring applications in human and veterinary medicine. Furthermore, key technical challenges, standardization issues, and regulatory considerations are discussed, offering readers a clear roadmap for advancing both foundational studies and translational applications of OoC systems.

## 1. Introduction

The concept of organ-on-chip (OoC) systems emerges at the intersection of two key ideas: the “organ,” representing the complex cellular architecture and physiological functions of tissues, and the “chip,” a microfabricated platform designed to replicate and control these biological processes in vitro. Ex vivo and in vitro models such as 2D cell lines, Transwell inserts, tissue explants, and spheroids have long provided invaluable insights into cellular behavior [1]. The introduction of organoid culture, exemplified by Sato’s intestinal organoids, marked a significant advance, offering self-organizing, three-dimensional structures that more closely mimic native tissues [2]. Yet, organoids face their own challenges, including variability in size and shape, difficulty in maintaining defined spatial arrangements for long-term analysis, limited accessibility to luminal compartments, and dependency on 3D scaffold support. Many conventional 3D models also lack multiscale organization and tissue–tissue interactions such as vascular endothelium communication with parenchymal and connective compartments, which is necessary for physiological relevance, and they often fail to replicate mechanical forces like fluid shear stress, tension, and compression [3,4]. These limitations hinder the study of intercellular communication, pharmacokinetics, and disease processes.

Microfluidic OoC platforms provide an opportunity to tackle these challenges. They offer precise control over fluid flow, gradients, and shear stress at microscale dimensions and enable efficient nutrient delivery and waste removal [5,6]. It also can replicate organ function and organ-organ communication in vitro by cellular confinement and physiologically relevant compartmentalization, allowing the formation of native tissue-like architecture with defined size and spatial organization. Rapid prototyping techniques such as PDMS soft lithography, micro-milling, and 3D printing allow researchers to design customized devices tailored to specific organoid systems and make microfluidic chips that permit partial compatibility with existing laboratory infrastructure [7,8,9]. Importantly, these platforms enable the modeling of sequential multi-organ processes, such as the absorption, distribution, metabolism, and excretion (ADME) of orally ingested substances, as well as endocrine and hormonal feedback loops critical for systemic physiology [10,11,12].

These advances are relevant regarding recent regulatory changes. In April 2025, the U.S. Food and Drug Administration (FDA) announced a phased plan to prioritize non-animal testing methods including the use of OoCs, organoids, and computational models for drug evaluation [13]. This initiative builds on the FDA Modernization Act 2.0 (2022), which removed the legal requirement for animal testing in certain applications and reflects growing confidence in New Alternative Methods (NAMs) to predict human-specific responses. Although challenges remain in validating NAMs for regulatory adoption, early demonstrations, such as the use of patient-derived intestinal organoids to predict the off-tumor toxicities of T cell-engaging bispecific antibodies, highlight the potential of these systems to complement or even replace traditional animal models [14].

**Figure 1:**
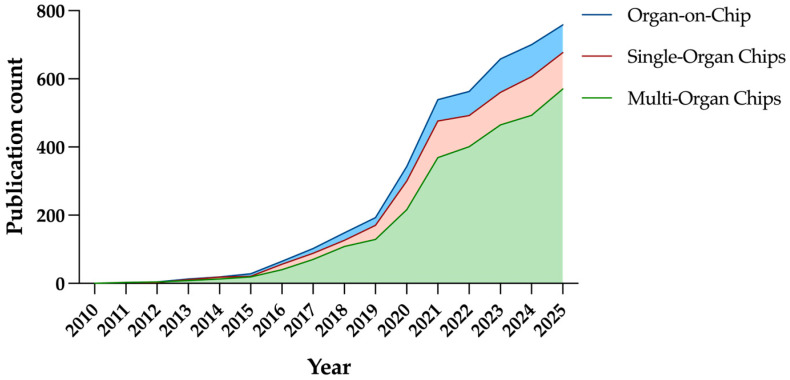
Publication output of organ-on-chip, single-organ chips, and multi-organ chips in past 15 years. (Data represent number of PubMed-indexed publications retrieved using queries ‘Organ-on-chip’, ‘Single-Organ Chips’, and ‘Multi-Organ Chips’).

Beyond regulatory and translational considerations, OoC provides unique opportunities for experimental biotechnology. Over the past decade, public investment and publication output in OoC research have risen sharply (Figure 1). As of 2025, nearly 800 publications have been published in the OoC field. The European Union has supported large-scale Horizon Europe projects such as UNLOOC with an overall budget of 70 million, while the National Institutes of Health (NIH) awarded USD 35.5 million to “Clinical Trials on a Chip” in 2020. Following the pioneering development of the lung-on-a-chip, numerous OoC systems have been engineered to replicate key structural and functional aspects of various human organs [15]. These platforms have progressed from single-organ models to integrated multi-organ and body-on-a-chip systems capable of simulating inter-organ communication [10]. 

This review focuses on the integration of organoids into microfluidic platforms, highlighting trends and advances in both human and veterinary OoC systems. It introduces readers to a range of topics from foundational concepts in microfabrication and perfusion flow to the practical construction of OoC systems. Meanwhile, it emphasizes key methodologies, technical considerations, and common challenges, offering a roadmap for researchers seeking to harness organoid-based microfluidics for basic and applied research. By bridging the strengths of organoids with the versatility of microfluidics, these integrated platforms can model human and animal physiology, disease, and drug responses with unprecedented fidelity.

## 2. Advances in Organoid Technology

Organoids are derived from stem cells, and can replicate key features of tissues and organs, providing a more authentic alternative to traditional cell cultures and animal models. This section outlines the key developments underlying organoid generation and application, including stem cell sources, culture methods, long-term maintenance, directed differentiation, and extracellular matrix scaffolds. Furthermore, it addresses current efforts toward organoid characterization and standardization which are critical steps to ensure reproducibility, tissue fidelity, and translational relevance across human and veterinary systems.

### 2.1. Derivation and Culture Methods

Organoids are typically generated from pluripotent stem cells or adult stem cells, which, under defined culture conditions, differentiate and organize into miniature organ-like systems. Advances in culture methods, including the use of extracellular matrix scaffolds, growth factors, and bioreactors, have significantly improved the efficiency and fidelity of organoid formation.

#### 2.1.1. Adult Versus Pluripotent Stem Cell-Derived Organoids

Adult stem cell (ASC) organoids are generated by isolating epithelial stem cells from biopsies or surgical tissue and embedding single cells or small fragments in an extracellular matrix to support three-dimensional growth and self-organization [16]. The first reliable demonstration using human colonic crypts showed that a single Lgr5-positive cell can regenerate a crypt–villus structure that can be passaged for months while retaining donor-specific mutations and epigenetic marks [17]. Tissues from dogs, cats, and pigs now follow the same process, providing species-specific platforms for veterinary gastroenterology, nutrition, and infection research [18,19,20]. Because the source cells are already committed to a tissue lineage, ASC organoids retain adult metabolic functions, drug transporter profiles, and injury responses [21,22]. These models are well-suited for studying toxicology as well as pharmacokinetics and pharmacodynamics. Compared to commonly used rodent models, they often provide a more relevant system due to their closer physiological similarity to humans, as well as comparable size and environmental conditions [23]. Although adult stem cell (ASC) organoids offer powerful models, they have several limitations. ASCs can only differentiate into a limited range of cell types related to their tissue of origin, and they may not give rise to all parenchymal cell types. For instance, adult pancreatic stem cells are typically ductal progenitors, and it is possible for them to differentiate to an endocrine lineage but not acinar cells [24]. In addition, the availability of primary tissue is often limited. Repeated passaging can also introduce biases, as cells with faster growth or survival advantages tend to dominate the culture, reducing the overall cellular diversity within the organoid population [25].

Pluripotent stem cell (PSC) organoids derived from embryonic or induced PSCs (iPSCs) close these gaps by tracking developmental signals that mimic gastrulation and organ specification in vitro. Directed protocols generate definitive endoderm and organ-specific progenitor cells and, after embedding in a matrix, form intestinal tubes containing mesenchyme and enteric neurons [26]. PSC strategies have been extended to iPSC lines from dogs and cattle, enabling the genetic manipulation of disease alleles and the modeling of inherited diseases affecting both companion animals and humans [27,28]. PSC organoids allow for access to embryonic stages and lineage communication but exhibit higher batch variability and the risk of residual undifferentiated cells. Current development efforts include patterned signaling gradients, inducible lineage reporters, and single-cell quality control to reduce heterogeneity and accelerate maturation [29,30]. Together, ASC and PSC organoids offer complementary tools: ASC cultures capture adult tissue behavior for drug screening and personalized therapies, while PSC organoids provide insights into congenital diseases and early toxicities, thus improving food safety and One Health prevention [26,31].

#### 2.1.2. Extracellular Matrix (ECM) Scaffolds for Organoid Culture

The self-assembly of organoids depends on a supportive extracellular matrix that provides adhesive ligands, mechanical resistance, and spatial containment. For more than a decade, Matrigel, a laminin-rich basement membrane extract, has been the standard scaffold because it reliably supports crypt formation and long-term expansion across epithelia [32]. However, Matrigel is undefined, variable, and derived from a mouse sarcoma, creating regulatory barriers for veterinary and human therapeutic applications [33]. Synthetic hydrogels made of polyethylene glycol (PEG) therefore allow for the independent tuning of stiffness, degradability, and ligand density [34]. A pioneering study demonstrated that a PEG network functionalized with laminin-111 and Arginine–Glycine–Aspartic acid (RGD) peptides supported the renewal and differentiation of intestinal stem cells without animal components and altered crosslinking density-controlled crypt formation. Subsequent work introduced protease-cleavable linkers and light-sensitive chemistry, enabling dynamic remodeling, custom pattern formation, and in situ stiffness gradients. Tailor-made viscoelasticity in PEG hydrogels improved epithelial migration and barrier closure in models of colon injury [35]. Hybrid strategies combine a decellularized tissue-specific ECM with synthetic backbones, combining native biochemical cues with mechanical stability, and have improved bile duct polarity and vascular ingrowth in liver organoids [36]. In addition to PEG, self-assembling peptide gels and plant-based polymers are being explored as xeno-free, cost-effective alternatives for the large-scale production of bovine organoids for vaccines, where the use of an animal-derived ECM is not possible [37,38].

#### 2.1.3. Long-Term Maintenance and Directed Differentiation

Maintaining organoids over dozens of passages while preserving proliferative capacity, genomic integrity, and multilineage differentiation requires the careful regulation of niche signaling and culture logistics. For instance, early intestinal crypt cultures could only be maintained short-term because Wnt3a factor, while essential for proliferation, on its own, was insufficient to preserve stemness. By adding epidermal growth factor (EGF), Noggin, and R-spondin1, long-term, self-renewing intestinal organoids were established. They maintained both proliferation and differentiation simultaneously [32]. This combination of factors recapitulates essential niche signals activating Wnt/β-catenin and bone morphogenetic protein (BMP) pathways, driving stem cell renewal and balanced lineage specification. Additional components such as IGF1 and FGF2 have been incorporated into refined culture media to simultaneously support stem cell expansion and lineage specialization as demonstrated in canine intestinal organoids [18].

Aside from intestinal organoids, liver organoids derived from canine or livestock tissues have demonstrated promising stability and functional maturation when maintained in culture with defined media formulations. Key components except EGF and Noggin often include hepatocyte growth factor (HGF), fibroblast growth factor (FGF), dexamethasone, and gamma-secretase inhibitor DAPT. The removal of R-spondin-1 is often necessary to push organoids out of a proliferative state towards maturation into hepatocytes [39]. Long-term cultures have been shown to retain liver functions, including albumin secretion, cytochrome P450 activity, and bile acid transport. A clear example can be found in feline liver organoids, which have been maintained with stability for up to 32 passages [40].

Another example can be airway organoids, featuring a respiratory epithelium including basal, ciliated, goblet, and club cell populations. They can be maintained in culture for over 1 year while preserving multilineage differentiation and physiological functions such as mucociliary clearance and response to airway toxins. The culture media for airway organoids combine factors supporting basal stem cell expansion (EGF, FGF2) with components promoting differentiation like Notch inhibitors or retinoic acid. The modulation of Wnt signaling balances progenitor self-renewal with maturation into specialized epithelial cell types [41].

While we have emphasized intestinal, liver, and airway organoids more, it is important to recognize that the field encompasses an ever-expanding diversity of organoid types derived from nearly every tissue and organ system. Organoids representing the brain, kidney, pancreas, mammary gland, bladder, reproductive tract, and many other tissues have been developed across multiple species, including humans and veterinary animals [42,43]. These organoids continue to evolve with advancements in culture techniques and differentiation protocols, enabling an increasingly faithful recapitulation of organ-specific structure and function in vitro.

In veterinary organoid research, human or murine-derived growth factors are frequently used but do not always optimally support their growth and differentiation due to species-specific biological differences. Ad hoc culture media proves to be essential for the generation of physiologically relevant organoids; for instance, chicken intestinal organoids exhibit significantly improved survival when cultured with chicken-derived RSPO1 and WNT3 compared to mammalian-derived factors [44].

Maintaining organoid cultures within defined size limits and passaging them at appropriate intervals are critical to preserving viability and phenotypic stability. As organoids grow to over an optimal size, diffusion limitations lead to central necrosis, compromising culture integrity. Delayed and extended passaging can also contribute to genetic and epigenetic drift, reducing reproducibility and physiological relevance [45]. Additionally, freeze–thaw techniques using DMSO-free cryoprotectants preserve expansion potential and drug efficacy profiles, which enables the effective biobanking of valuable genetic material from endangered species or elite breeding lines [46]. Reliable cryopreservation pipelines also allow for organoid maintenance over extended periods, reflecting chronic disease progression and long-term drug exposure, which is crucial for translational veterinary research.

### 2.2. Organoid Characterization and Standardization

The standardized characterization of organoids is essential to ensure reproducibility, tissue fidelity, and translational relevance, especially when adapting protocols across species for veterinary applications. Morphological accuracy is assessed by phase-contrast and immunofluorescence microscopy. In human and murine models, organoids reproduce tissue-specific structures such as crypt–villus axes in intestinal cultures, hepatocyte zonation in liver organoids, and cortical stratification in brain organoids [47]. In veterinary research, similar phenotypic characteristics have been validated for organoids from dogs, cats, cattle, and horses, confirming the conservation of architecture, cell polarity, and cell type composition in the gastrointestinal, hepatic, and renal systems [43]. However, variations in tissue sampling, ECM composition, and passage conditions lead to morphological heterogeneity, necessitating the need for cross-laboratory benchmarks.

Molecular fidelity is assessed using transcriptomic and proteomic profiling, comparing organoids to their tissue of origin. Single-cell RNA sequencing (scRNA-seq) has become a golden standard, resolving epithelial subtypes and developmental trajectories [48]. In species like dog and pig, where reference genomes and cell-type atlases are still developing, scRNA-seq enables the de novo classification of lineages and detection of culture-induced drift. Veterinary intestinal and liver organoids have shown a stable expression of key lineage markers, drug metabolism enzymes (e.g., CYP450s), and epithelial tight junction proteins over multiple passages [49]. Proteomic approaches, including data-independent acquisition (DIA), data-dependent acquisition (DDA), label quantification (LQ), and label-free quantification (LFQ) add post-transcriptional resolution, capturing stress responses and post-translational modifications absent in RNA-based assays.

Batch-to-batch variability remains a major challenge, amplified by differences in donor age, health status, tissue ischemia, and Matrigel composition. This is relevant in veterinary practice, where donor access may be opportunistic (e.g., post-euthanasia) and freezing protocols are inconsistently applied. Tools such as live-cell metabolic assays, transepithelial electrical resistance (TEER) measurements, and barcoding strategies help assess consistency and performance in real time. Advanced imaging techniques, including confocal, light-sheet, and high-content microscopy, support automated morphology tracking and drug response profiling [50], now applied to organoids in high-throughput toxicity screens [51,52].

### 2.3. Emerging Trends

Three rapidly developing fields are transforming organoid research and expanding its impact on veterinary medicine: CRISPR-based genome editing, patient-derived organoids, and living biobanks. CRISPR-Cas9 technology enables the precise and scar-free modification of endogenous loci in organoids, enabling both gain- and loss-of-function studies in a controlled, tissue-specific context. The sequential editing of tumor suppressors and oncogenes in human intestinal organoids reconstructed stepwise colorectal tumorigenesis and revealed genotype–drug interactions [53]. Similar approaches corrected the CFTR F508del mutation in intestinal organoids from cystic fibrosis patients, restored chloride transport, and predicted individual responses to modulators [54].

Patient-derived organoids (PDOs) capture the genomic, epigenomic, and microarchitectural features of individual tumors or diseased tissues. Large PDO panels for colorectal cancer have reproduced clinical chemosensitivity patterns, enabling functional precision oncology within two weeks of biopsy [55]. Living organoid biobanks institutionalize these efforts by standardizing collection, expansion, quality control, and data integration. The first multicenter human colorectal biobank demonstrated that cryopreserved lines retain their genomic accuracy and drug response phenotypes after global distribution [56]. This was followed by collections of breast, pancreas, and respiratory tissue, each linked to clinical annotations and multi-omics dashboards [57]. Veterinary equivalents are emerging: the Dog K9Org Biobank and the PigBiobank catalog, comprising normal and diseased tissue from different breeds, age groups, and geographic regions [58,59]. These repositories accelerate cross-study reproducibility, lower barriers to entry for new labs, and create reference sets for single-cell atlases, proteomic benchmarks, and machine learning classifiers. By converging CRISPR editing, PDO workflows, and open biobanks, the field is moving toward rigorous, species-specific platforms that support precision medicine in human and veterinary medicine while reducing reliance on animal testing.

## 3. Microfluidics and Organ-on-a-Chip Platforms

Microfluidics and OoC platforms combine engineering with biology to better mimic physiology. Using microscale channels and chambers, these systems control fluid flow and cellular environments, enabling the recreation of organ-level functions.

### 3.1. Fabrication of Organ-on-Chip

**Figure 2:**
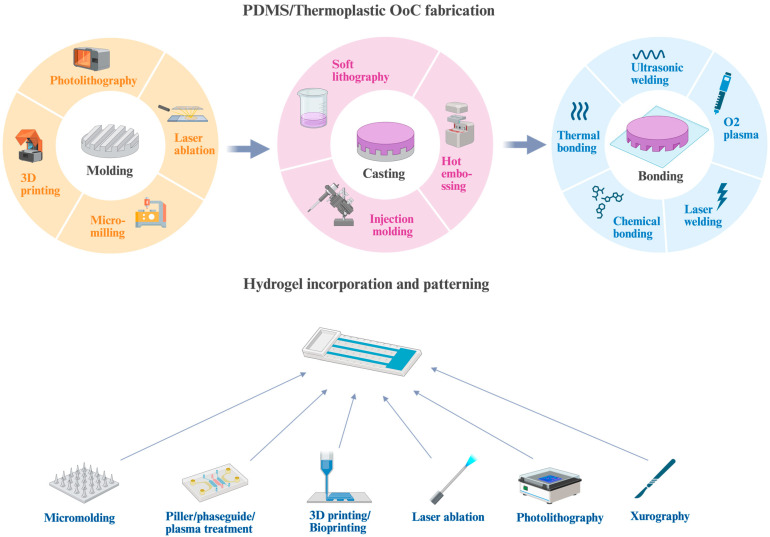
The fabrication of organ-on-chip (OoC) devices. Molding is achieved using methods such as 3D printing, photolithography, micro-milling, or laser ablation. Once the mold is prepared, channels are cast in PDMS or thermoplastics. For proof-of-concept studies, soft lithography is commonly used, while hot embossing and injection molding are preferred for large-scale production. To create closed microchannels, the patterned layer is bonded to PDMS, thermoplastic, or glass substrates using techniques such as oxygen plasma treatment, the use of double-sided adhesives, thermal or chemical bonding, laser welding, or ultrasonic welding. Hydrogels are integrated into the chip and patterned to define channels and chambers. Several micropatterning strategies can be applied, including micromolding, pillar/phaseguide structuring, plasma treatment, laser ablation, 3D printing or bioprinting, photolithography, and xurography. Created with BioRender.com.

The concept of microfabrication originated from the semiconductor industry and was coined in the 1950s. Since Jean A. Hoerni invented the planar manufacturing process in 1959, microfabrication has developed rapidly. It enables the fabrication of features at the micron to nanometer scale and allows entire systems to be built on a chip [60]. Later, this concept was implemented in biological research, where one could engineer tissues or organoids to mimic physiological conditions in vitro. Below we discuss commonly used OoC devices according to the primary material used in their fabrication (Figure 2).

#### 3.1.1. PDMS-Based OoC

Polydimethylsiloxane (PDMS) is a silicone-based elastomer that has become one of the most widely used materials in microfluidics and bioengineering. Its popularity stems from a combination of favorable properties including biocompatibility, optical transparency, flexibility, gas permeability, and a relatively simple and cost-effective fabrication process.

The most common fabrication method for PDMS-based microfluidic devices is soft lithography, a process in which PDMS is cast over a structured mold to replicate microchannel features. Two main approaches are used to create these molds: photolithography using SU-8 photoresist and 3D printing.

In the photolithography-based method, SU-8, a negative photoresist, is first spin-coated onto a clean silicon wafer. The spin speed determines the thickness of the coated layer, which is crucial for defining the height of the final microchannels. The coated wafer is then exposed to ultraviolet (UV) light through a photomask that contains the desired pattern. Upon exposure, the UV light crosslinks SU-8 in the exposed regions, making it resistant to the developer solution. After development, the unexposed areas are washed away, leaving behind a raised pattern that serves as a master mold for PDMS casting [61]. Sub-micron feature size can be achieved using this method [62].

Alternatively, 3D printing has emerged as a fast and highly customizable method for mold fabrication. Desktop-grade vat polymerization techniques like stereolithography (SLA) and Low Force Display™ (LFD), and the SLA variant is commonly used to produce OoC molds. They are limited to a minimum feature size of approximately 50 μm and can have a surface roughness that affects perfusion flow dynamics [63]. In contrast, using Projection Micro Stereolithography (PµSL) (Boston Micro Fabrication (BMF)), such as via microArch^®^ S230, can achieve resolutions as fine as 2 µm. This advanced capability allows 3D-printed molds to incorporate complex geometries, multi-height structures, and smooth surfaces that significantly reduce the need for postfabrication steps such as port punching, bridging the gap between rapid prototyping and high-fidelity production.

Although PDMS offers many advantages, it also presents certain challenges that must be addressed depending on the application. One major limitation is its hydrophobicity, which can hinder wettability and cell adhesion. To overcome this, surface treatments are often employed. Oxygen plasma treatment temporarily increases surface hydrophilicity, but its effect diminishes over time due to hydrophobic recovery. More permanent modifications may involve chemical coatings such as fibronectin, collagen, or poly-D-lysine to enhance cell adhesion and compatibility for long-term biological studies [64]. Another drawback is related to applications such as pharmacological testing or high-throughput screening. The material’s tendency to absorb hydrophobic small molecules can lead to the unpredictable loss of drugs, dyes, or signaling compounds, thereby compromising the accuracy of toxicity assays and pharmacokinetic/pharmacodynamic (PK/PD) studies [65]. In addition, PDMS is also incompatible with many organic solvents, which can cause swelling, delamination, or even complete device failure. These material limitations have prompted a growing interest in alternative substrates such as thermoplastics [66].

#### 3.1.2. Thermoplastic-Based OoC

Thermoplastics are polymeric materials that soften when heated and harden when cooled, making them suitable for molding and mass manufacturing. In OoC applications, they offer a rigid, biocompatible, and scalable alternative to PDMS, especially when drug interaction and reproducibility are critical. There are preferably transparent thermoplastic polymers such as polystyrene (PS), polymethyl methacrylate (PMMA), polycarbonate (PC), and cyclo-olefin copolymers (COCs), and cyclo-olefin polymers (COPs) are used to fabricate microfluidic chips [67].

However, thermoplastics require different fabrication techniques compared to PDMS. These methods are more suited for industrial-grade precision and volume manufacturing. Each process begins with the computer-aided design (CAD) of OoC devices, where the chip architecture is defined with high precision. Based on this design, a master mold is fabricated from durable materials such as stainless steel or nickel using advanced micromachining techniques. These may include high-resolution micro-milling, laser ablation, or lithography-based patterning followed by electroforming [68]. Following mold fabrication, thermoplastic chips are produced mainly using injection molding and hot embossing [69,70].

Once the molded component is produced, it requires bonding to a second layer, either a flat thermoplastic sheet or a membrane or coverslip to form enclosed microchannels. Several bonding strategies are available depending on the material properties and application requirements. Thermal bonding involves applying heat and pressure to fuse the surfaces, although care must be taken to avoid deforming delicate microfeatures. Solvent bonding uses chemical solvents or vapors to partially dissolve the surfaces for fusion, though it may leave residues that affect biocompatibility. Alternatively, UV-curable adhesives or double-sided adhesive films can be employed to join components while maintaining optical clarity. For highly automated or industrialized production lines, laser welding and ultrasonic welding are increasingly used to provide fast, strong, and reproducible bonds [71].

However, some challenges remain. The initial cost of mold fabrication is high, which can be a barrier for early-stage research or custom applications. Design constraints related to demolding angles and flow behavior must be considered during the CAD phase, and multilayer alignment requires high-precision tooling. In addition, thermoplastics have lower gas permeability than PDMS, which can limit oxygen exchange in certain cell culture setups unless compensated by the integration of porous membranes or gas-permeable regions [72]. Alternatively, styrene–ethylene–butylene–styrene (SEBS) thermoplastic elastomers combine both PDMS and thermoplastic benefits, which makes them highly suitable for OoC applications, balancing ease of prototyping, reproducibility, and scalability with essential mechanical flexibility and optical clarity [73,74].

#### 3.1.3. Hybrid OoC

OoC devices are frequently fabricated as hybrid systems that combine multiple materials. Common configurations include PDMS bonded to glass microscope slides, or integrated with porous polymer membranes for channel separation, or incorporating hydrogels to form 3D scaffolds. In this section, we focus on the application of hydrogel scaffolds in OoC fabrication.

Hydrogels are widely used in biomedical engineering due to their biocompatibility, high permeability to small molecules, and optical transparency. Broadly, hydrogels can be categorized as either natural (e.g., gelatin, silk fibroin, collagen) or synthetic (e.g., polyvinyl alcohol (PVA), polyethylene glycol (PEG)) [75]. Natural hydrogels offer inherent advantages such as biocompatibility, biodegradability, and low cytotoxicity, though they lack tunable mechanical properties. Consequently, natural hydrogels are often combined with synthetic polymers to enhance structural stability while maintaining bifunctionality [76].

Hydrogel hybrid OoC platforms provide hydrated, cell-friendly environments that closely resemble the native extracellular matrix (ECM). This makes them well-suited for modeling soft tissues such as the brain [77], liver [78], and skin [79], as well as tissue barriers, like the gut [80] and blood–brain barrier [81], and complex vascular networks [82]. To incorporate hydrogels into chip architectures, devices are often engineered with ridges, membranes, or pillar structures that anchor the hydrogel, prevent collapse under perfusion, and maintain spatial definition. A representative example is the OrganoPlate^®^ platform developed by Mimetas, which uses PhaseGuide™ ridge structures to enable membrane-free compartmentalization and perfused 3D cultures, supporting the co-culture of distinct yet interacting cell populations [83]. In other cases, porous and fibrous scaffolds such as electrospun membranes are integrated to stabilize hydrogels and facilitate their patterning with techniques like xurography. Using this approach, hydrogel features as small as 200 µm can be fabricated at scale [84]. Alternatively, plasma surface treatment can be used to generate localized hydrophilic regions on otherwise hydrophobic substrates, thereby enabling the precise placement of hydrogels without relying on rigid geometric confinement [85].

In addition to the techniques mentioned above, a wide range of micropatterning strategies have been adapted for hydrogel integration in OoC systems. For instance, PDMS microstamps can imprint defined geometries into hydrogels, thereby guiding tissue organization by creating spatially controlled microenvironments [86]. Another powerful approach is photopolymerization, where UV-crosslinkable hydrogels are patterned via photomasks, uniform illumination, or direct laser writing to create high-resolution microstructures [87]. However, this method is restricted to UV-curable polymers and often requires labor-intensive post-processing to remove unpolymerized precursors. Complementing these, laser-based techniques such as femtosecond laser ablation enable the direct sculpting of hydrogel surfaces or microscale architectures within conductive or bioactive composites, further broadening design possibilities [88,89]. Building on these precision methods, molding and photo-thermal polymerization have been integrated with 3D printing to streamline and standardize the large-scale fabrication of hydrogel microstructures [90]. Bioprinting represents an even more advanced frontier, as it allows for the direct embedding of living cells into hydrogel matrices with exceptional spatial accuracy, facilitating the construction of complex and functional OoC models that more faithfully recapitulate native tissue architecture [91,92].

Despite their many advantages, hydrogels remain underutilized in microfluidic and OoC devices. Their relatively low stiffness presents challenges for microfabrication and limits their structural integrity during long-term experiments, constraining their broader adoption in research applications [93].

A comparative summary of the merits and limitations associated with the three different types of OoCs is presented in Table 1.

### 3.2. OoC Design Strategies

In this section, we discuss organ-on-chip design strategies according to compartment separation and biological question-driven considerations, rather than organ type. For more comprehensive reviews focusing on specific organ models, readers are referred to other excellent reviews [94].

Organs are inherently multicellular systems, in which parenchymal cells perform the main tissue-specific functions, but require the support of stromal, endothelial, and immune cells to mature and maintain physiological activity. In addition, vascularization and nutrient supply are essential factors to ensure long-term viability and proper tissue function. Therefore, recreating organ-level physiology on a chip requires not only the inclusion of different cell types but also their appropriate spatial organization and functional separation. A central challenge in chip design is the achievement of the co-culture of heterogeneous cell populations while maintaining physiologically relevant compartmentalization that allows for selective communication.

To address this, a variety of strategies have been developed to physically or functionally separate cell populations within microfluidic systems while still enabling biochemical crosstalk. These strategies broadly include (i) microchannel-connected cultures, in which adjacent organoid or tissue compartments are linked through perfusable channels; (ii) porous membranes, which establish discrete yet permeable barriers between cell layers; and (iii) hydrogel-based channels or barriers, which combine separation with extracellular matrix (ECM)-like support. Each of these approaches presents unique advantages and limitations in terms of biomimicry, mechanical stability, and compatibility with long-term culture (Table 2). In the following, we discuss these designs in detail.

#### 3.2.1. Microchannel-Connected OoC

One approach to achieving cell-type separation while maintaining functional communication is to integrate organoids with interconnected microchannels. In this design, organoids are placed in adjacent chambers linked by microchannels, enabling the exchange of soluble factors and metabolites while maintaining spatial separation between cell populations. Such setups are valuable for modeling developmental processes or diseases, such as heart-on-chip systems [95], where a continuous supply of oxygen and nutrients is critical. They are also ideal for studying inter-organ interactions, including the coupling of parenchymal organoids with supporting endothelial or stromal compartments [96], liver–pancreas crosstalk [97], or multi-organ systems [98,99]. The key advantage of this approach is its ability to maintain physiologically relevant gradients that drive tissue organization and function. However, challenges remain, especially in ensuring uniform organoid size and establishing robust barrier functions across compartments.

#### 3.2.2. Porous Membrane OoC

Porous membranes have been widely utilized in OoC systems to establish barrier functions that separate different cell types while allowing for selective molecular exchange. These membranes enable the recreation of epithelial–endothelial interfaces, facilitating studies on cell–cell communication, barrier integrity, and tissue response to mechanical stimuli.

Conventional porous membranes in OoCs are often fabricated from materials like polycarbonate (PC) and polyethylene terephthalate (PET), which are commercially available with defined pore sizes, biocompatibility, and transparency [100]. These materials have been extensively applied in modeling the blood–brain barrier [101], intestinal barrier [28], and alveolar–capillary interface [102] and connecting multi-organ-on-chip platforms [103]. A recent study introduced a liver-on-chip model incorporating hollow fiber membranes (HFMs)—3D tubular structures fabricated from porous polyethersulfone (MicroPES). These membranes enable the establishment of polarized epithelial monolayers with two independently perfusable microfluidic compartments. Compared to conventional flat membranes, HFMs provided a more physiologically relevant architecture and significantly promoted cellular differentiation [104].

However, the rigid nature of these membranes may restrict mechanical stimulation. To overcome this, PDMS-based stretchable membranes have been developed such as those incorporated in the Emulate Chip-S1. These flexible membranes replicate dynamic forces present in vivo, including shear stress from blood flow and the peristaltic-like motions of the intestine, enhancing the physiological relevance of the model, especially in terms of the intestines and lungs [105]. Another innovative approach involves the use of biological membranes composed of collagen and elastin, as demonstrated in a second-generation lung-on-a-chip model [106]. This model utilizes a stretchable and biodegradable membrane made from these extracellular matrix proteins to emulate an array of tiny alveoli with in vivo-like dimensions. The collagen–elastin membrane outperforms PDMS in several ways: it does not absorb small molecules, is biodegradable, and can be easily tuned to modify its thickness, composition, and stiffness. This biological membrane supports the culture of primary human lung alveolar epithelial and endothelial cells, preserving barrier properties for extended periods and providing a more accurate representation of the lungs’ air–blood barrier.

**Table 2:** A comparison of three design strategies in OoC.

	Application	Limitation	Ref.
Microchannel 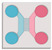	– Inter-organ interactions – Spatial separation – Physiological gradients	Lack of barrier function	[95,96,97,98,99]
Membrane 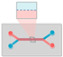	– Barrier function – Cell interface – Mechanical stimuli	No recreation of complex 3D structure	[100,101,102,103]
Hydrogel 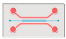	– Model complex tissue structure – Angiogenesis/invasion – Natural diffusion gradient	Long-term structural stability proves to be difficult	[107,108,109]

#### 3.2.3. Hydrogel OoC

Hydrogel-based compartments offer a more biomimetic approach to cell separation within organ-on-chip systems. Hydrogels can serve as both physical barriers and ECM-like scaffolds that support cell attachment, migration, and differentiation. By patterning hydrogels as channels or barriers, multiple cell types can be cultured in close proximity with the controlled diffusion of signaling molecules across the hydrogel. For example, a study incorporating plasma-treated, pillar-free hydrogel channels enables a biomimetic blood–brain barrier [107], while a bilaterally accessible hydrogel barrier intestinal chip allows for the independent manipulation of the apical and basal sides [108]. This strategy is very useful for studying stromal–parenchymal interactions and vascularization processes, as hydrogels can be engineered to incorporate growth factors, gradients, or degradable components [109]. As mentioned above, the main challenge lies in maintaining the long-term structural stability of hydrogels under continuous flow conditions.

Based on the advantages and limitations of different microfluidic compartment separation strategies discussed above, the choice should be guided by the specific biological question under investigation, whether the focus is on mechanical or biochemical function, barrier properties, organ development or disease modeling, or the integration of single- versus multi-organ systems. Once the design strategy is defined, CAD tools can be used to create the chip layout, which is then fabricated accordingly. The organoids are subsequently integrated into the device, followed by the initiation of perfusion to establish dynamic culture conditions.

### 3.3. Perfusion Methods and Flow Simulation

Perfusion and flow control are crucial elements that distinguish OoC systems from conventional static cultures. Microfluidic flow mimics physiological shear stresses, improves nutrient and oxygen delivery, and enables long-term culture by removing waste products. Controlled flow supports tissue polarization, drives morphogen gradients, and enables dynamic exposure to drugs or pathogens [110,111].

In OoC systems, perfusion can be created by active pumping or passive flow manipulation. Active methods utilize a mechanical force to generate flow. The most used types of pumps in OoC include syringe pumps, peristaltic pumps, pressure-driven systems, and micropumps. Each pump type presents distinct advantages and limitations (Table 3).

Syringe pumps are popular for their high precision and ease of use, operating by driving a plunger at controlled speeds to dispense liquid from a syringe. They excel in applications needing accurate, pulsation-free flow at low volumes, such as drug delivery, gradient generation, or short-term microfluidic experiments. However, their capacity is limited by syringe size, restricting use in long-term or recirculating setups, and frequent refilling is needed for multiple inlets or continuous flow [112]. In contrast, peristaltic pumps employ rotating rollers to compress flexible tubing, propelling fluid without direct contact to minimize contamination and enable easy switching or cleaning. They are ideal for recirculating systems, like OoC devices requiring extended perfusion. Drawbacks include inherent pulsatile flow (unless dampened), lower precision at low rates compared to syringe or pressure systems, and tubing degradation that affects stability and requires maintenance [113]. Pressure-driven pumps provide precise, responsive fluid control by using regulated gas pressure to push fluid from reservoirs into devices. When paired with flow sensors and feedback loops, they enable high-resolution, real-time flow modulation, making them suitable for complex tasks like multichannel setups, dynamic profiles, and high-throughput screening. However, they involve more complex setups, require a compressed gas source (e.g., air or nitrogen) that raises costs, and demand calibration for factors like viscosity and channel resistance, especially with heterogeneous or biological fluids [114,115]. Mechanical micropumps incorporate membranes, pistons, or valves actuated by piezoelectric, electrostatic, or thermopneumatic forces to actively displace fluid at nano- to microliter rates per minute. For example, piezoelectric versions use vibration-driven membrane deflection for rapid response and fine control [116]. Unlike bulky external systems, these can be integrated directly into microfluidic chips for compactness, localized control, and automation. For instance, a study presents a disk-shaped organ-on-chip platform with integrated peristaltic micropumps that eliminate external tubing, enabling parallel endothelial cells to maintain long-term viability and support physiologically relevant assays such as cytokine stimulation and whole-blood perfusion [117]. Limitations include costly and complex fabrication, difficulties with integration, restricted flow ranges, susceptibility to clogging or biofouling, and reliability concerns in long-term biological use [118].

There is a growing interest in pumpless and tubeless organ-on-chip systems due to their simplicity and reduced reliance on external equipment. A common approach is gravity-driven passive flow [119], which relies on the height difference between fluid reservoirs to generate hydrostatic pressure that drives liquid through microchannels. The pressure difference can be described as ΔP = ρgΔh, where ρ is fluid density, g is gravitational acceleration, and Δh is the vertical height difference between inlet and outlet reservoirs [120]. For instance, a microfluidic vessel-on-chip platform employing gravity-driven bidirectional flow via rocking was demonstrated in a STAR Protocols study [121]. In a different report, a pumpless recirculating OoC utilized gravity-driven unidirectional flow with a thermoplastic microfluidic design to support endothelial cells, liver organoids, and circulating immune cells, enabling multi-tissue integration without the need for external pumps or tubing [122]. Gravity-driven systems provide steady, though generally low flow rates without the need for external pumps or power sources, making them attractive for low-resource environments or settings where mechanical components increase contamination risks or operational complexity. However, flow rates depend on reservoir levels and thus vary over time unless leveling or feedback mechanisms are incorporated. Moreover, the precise control of flow profiles and switching remains limited compared with active pumping strategies such as syringe or pressure-driven systems. Nonetheless, gravity-driven flow continues to be widely adopted in proof-of-concept and educational applications.

Another pumpless approach employs hydrophilic thread-driven evaporation to generate tunable flow, enabling physiologically relevant shear stress and supporting long-term intestinal and lung organoid cultures comparable to those maintained with precision pumps [123].

Microfluidic flow behavior is governed by fundamental parameters including medium density and viscosity, flow rate, concentration gradients, and the Reynolds number. By adjusting these factors and optimizing microchannel geometry, researchers can control both the magnitude and direction of flow [124]. Still, establishing robust perfusion systems for the 3D culture of mammalian cells remains challenging. Design considerations include culture configuration, material choice, and channel layout, while operational challenges involve sterilization, efficient cell seeding, optimizing nutrient and oxygen transport under physiologic shear, and preventing bubble formation.

To address these issues, computational flow simulations using tools such as FLOW or COMSOL Multiphysics are increasingly employed. These platforms allow for the prediction of fluid dynamics, nutrient and oxygen distribution, and the virtual testing of chip designs prior to fabrication, reducing costly trial-and-error. For instance, in liver-on-chip models, COMSOL has been used to model oxygen gradients across hepatocyte cultures, optimizing channel geometry and flow rates to maintain physiologically relevant oxygenation—an essential factor for sustaining hepatocyte function over long-term culture [125].

## 4. Functional Readouts and Data Analysis

The utility of OoC systems is not only in replicating tissue architecture and physiology; their transformative potential also lies in coupling microengineered environments with integrated sensing and functional readouts. Without such capabilities, OoCs risk being reduced to sophisticated culture devices rather than analytical platforms [126]. Real-time, continuous, and quantitative monitoring transforms them into dynamic systems for probing tissue behavior, uncovering disease mechanisms, and evaluating therapeutic interventions with higher fidelity than static assays.

Biosensors are central to this evolution. Mechanical sensors not only quantify tissue deformation, shear stress, and contractility but also link microenvironmental forces to mechano-transduction pathways, which are increasingly recognized as drivers of disease progression and therapeutic resistance [127]. Electrochemical sensors expand this functional window by enabling the continuous monitoring of pH, oxygen, glucose, metabolites, and biomarkers, thereby providing direct insight into metabolic states and their fluctuations under stress or drug exposure. Optical sensors, including fluorescence- and luminescence-based modalities, add a noninvasive layer of observation, allowing for the spatiotemporal mapping of signaling events and structural remodeling [128]. Taken together, these modalities move OoCs outside of the limit of endpoint characterization toward a regime of biological dynamic systems in vitro.

However, integrating sensors into microfluidic platforms introduces a new set of bottlenecks. Signal drift, fouling from proteins or adherent cells, and flow perturbations caused by miniaturization can compromise accuracy. Sensor specificity further limits scalability, as devices optimized for one organoid or tissue type may not function reliably across others. Moreover, embedding multiple sensing modalities often requires sophisticated fabrication and specialized expertise, raising costs and limiting accessibility [129]. These challenges suggest that universal integration is not always the most practical path forward. Instead, there is growing momentum toward hybrid strategies that emphasize compatibility with standard laboratory infrastructure. For example, chips manufactured in multiwell formats with optical transparency and accessible channels can leverage existing imaging systems and high-throughput readers. Commercial platforms such as the Mimetas OrganoPlate show how standardization lowers adoption barriers, enabling reproducible functional assays without requiring custom instrumentation.

While such design strategies improve accessibility, they also increase the volume and complexity of data produced. High-dimensional imaging and multi-parameter readouts generate datasets that quickly outpace conventional analysis. Here, artificial intelligence (AI) and machine learning emerge as indispensable partners. Besides automating feature extraction, these tools uncover hidden phenotypic signatures and capture dynamic processes that human observers often miss. For example, deep learning applied to nuclear morphology has been shown to predict cellular senescence across tissues and species, establishing morphology as a biomarker of aging and age-related disease [130]. Similarly, the CellPhenTracker framework integrates phenotypic and metabolic information to trace lineage trajectories in tumor organoids, revealing how lactate regulates cancer stemness through epigenetic reprogramming [131]. These examples underscore the capacity of AI not merely to analyze but to redefine biological interpretation, turning OoCs into predictive models of human physiology and pathology.

**Figure 3:**
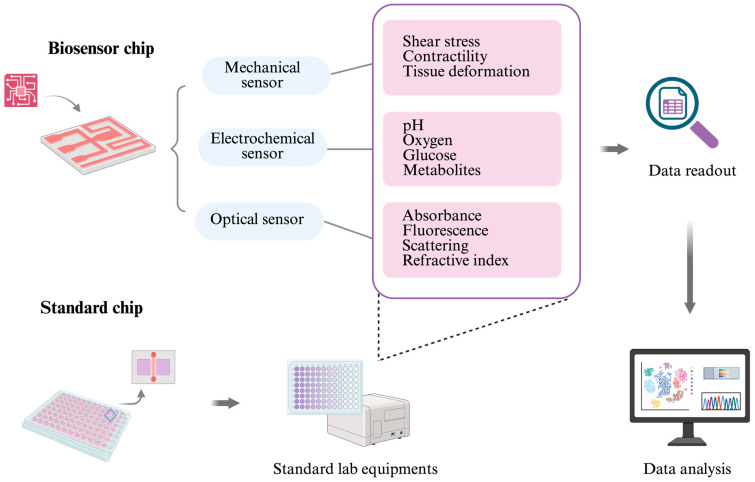
Biosensors can be integrated directly into OoC systems, or the chip can be designed to be compatible with standard laboratory equipment. Common sensor types include mechanical sensors (for measuring shear stress, tissue deformation, and cell contractility), electrochemical sensors (for monitoring pH, oxygen, glucose, and other metabolites), and optical sensors (for detecting absorbance, fluorescence, scattering, and refractive index changes). The large and complex datasets generated by these biosensors can be further analyzed using artificial intelligence (AI) tools.

Progress in this space is not only about engineering better chips but about embedding them within a broader ecosystem of functional monitoring and computational interpretation. By uniting tissue engineering, sensor technology, and advanced analytics, next-generation OoC platforms are positioned to deliver scalable, reproducible, and clinically relevant tools for precision medicine, drug discovery, and mechanistic biology (Figure 3).

## 5. Standardization and Quality Control (QC) in OoC Technologies

The advancement of OoC technologies is limited by the lack of standardized methodologies, resulting in substantial variability and reduced reproducibility across laboratories and platforms. A major contributing factor is the diversity of proprietary devices, materials, and readout systems developed by individual manufacturers. While such diversity drives technological innovation, it also introduces incompatibilities that hinder data comparability, integration, and regulatory validation. The absence of harmonized data formats, calibration procedures, and performance benchmarks further complicates the alignment of experimental outcomes across different systems [132].

To address these challenges, several international initiatives have been launched to promote standardization and reproducibility in OoC research. The ORCHID project (Organ-on-Chip in Development), funded under the EU’s Horizon 2020 program, produced the first European OoC roadmap and established the European Organ-on-Chip Society (EUROoCS), a multi-stakeholder network linking academia, industry, and regulatory agencies to facilitate data comparability and validation. At the global level, the International Organization for Standardization (ISO) is developing ISO/WD 25693, a biotechnology standard that outlines the design, fabrication, and evaluation of OoC platforms for substance testing, representing a crucial step toward international harmonization. The CEN/CENELEC Focus Group on Organ-on-Chip (FGOoC) highlights three key priorities to improve reproducibility, comparability, and reliability in OoC research and applications [133]. First, standardization is needed in cell sourcing, biomaterial formulation, and microfluidic materials and operational parameters to minimize experimental variability. For example, adopting well-characterized iPSC lines with documented passage history, alongside certified ECM formulations with defined stiffness and biochemical composition, improves consistency across laboratories [134]. Similarly, establishing standardized guidelines for material selection, flow rates, and shear stress conditions within microfluidic systems enhances physiological relevance and cross-platform comparability. This is particularly important given the wide range of substrates used in chip fabrication, such as PDMS, thermoplastics, and hybrid polymers which differ in gas permeability, surface properties, and small-molecule adsorption. Second, robust QC measures are essential throughout the OoC development and operation pipeline. QC checkpoints include verifying cell identity and purity via lineage-specific markers such as insulin and PDX1 for pancreatic β-cells, assessing barrier integrity using TEER in epithelial chips, and validating mechanical and fluidic performance through sensor calibration and leakage testing. Implementing traceable QC documentation ensures data integrity and supports regulatory compliance. Third, standardized experimental design principles are critical to ensure the accuracy, precision, and reproducibility of biological experiments. The FGOoC recommends the routine inclusion of positive and negative controls, for instance, hepatotoxins such as acetaminophen in liver-on-chip assays, along with adequate sample sizes and biological replicates to ensure statistical robustness. Blinded data analysis and the use of internal reference standards further minimize operator bias and enhance reproducibility.

Together, these measures provide a cohesive framework for cross-platform validation and regulatory acceptance, ultimately advancing the translational impact of OoC technologies in pharmaceutical, biomedical, and veterinary applications.

## 6. Future Perspectives and Challenges

Organoid and organ-on-a-chip technologies are advancing rapidly and holding high potential for reshaping biomedical research in both human and veterinary medicine. Yet, to move from proof-of-concept demonstrations to widely adopted and reliable tools, several critical scientific, technical, and regulatory challenges must be addressed.

A central future direction lies in the development of systems that better capture the complexity of living organisms. Current OoC models remain limited by the absence of functional vascular networks, neural innervation, and immune components, all of which are crucial for replicating dynamic tissue–tissue and organ–organ interactions. Incorporating vascularization would improve nutrient delivery and drug pharmacokinetics, while the addition of immune and stromal elements would allow for the study of processes such as inflammation, infection, and tumor–immune crosstalk [135,136]. Advances in stem cell engineering, biofabrication, and bioprinting are expected to drive this progress, but the challenge of controlling spatial and temporal integration across multiple cell types remains considerable [137,138].

Another important challenge is the need for standardization and scalability. Many current devices are handcrafted and operate at a small scale, limiting their reproducibility and applicability in high-throughput contexts such as drug discovery or toxicology testing [139]. The future of the field is likely to move toward modular, plug-and-play devices with interchangeable organoid inserts that allow researchers to tailor systems according to experimental needs. The automation of assembly, culture, and monitoring will be essential to achieve large-scale use, but reducing the cost and technical complexity of such platforms remains a significant obstacle.

In veterinary medicine, organoid and OoC technologies present unique opportunities and challenges. Species-specific systems, such as dog-on-a-chip, cow-on-a-chip, or pig-on-a-chip, could transform fields ranging from disease modeling and vaccine testing to food safety and drug residue monitoring [140,141,142]. However, their development is hindered by the lack of standardized protocols, reference datasets, and optimized differentiation methods for non-human species. Animals with distinctive physiological traits, such as the ruminant digestive system, pose additional hurdles for the design of physiologically relevant models. Addressing these gaps will be critical for realizing the full potential of OoC technologies in comparative and veterinary biomedical research. While animal studies remain indispensable in medical research, efforts are underway to accelerate the adoption of alternative methods.

Another promising avenue for the future is the integration of OoC systems with computational tools and artificial intelligence. High-content imaging and machine learning algorithms are already being applied to monitor organoid growth, predict differentiation trajectories, and quantify drug responses [143]. Coupled with physiologically based pharmacokinetic modeling, these tools could simulate whole-body drug distribution and metabolism across species. The challenge, however, lies in generating sufficiently robust datasets to train these models and ensuring interoperability between computational outputs and experimental data [144].

Cost and accessibility also remain persistent barriers. The high expenses associated with chip fabrication, biosensors, and specialized instrumentation limit adoption, particularly in veterinary institutions where infrastructure and resources are often constrained. Future perspectives include the development of low-cost microfabrication techniques, reusable chip designs, and simplified fluid-handling systems that reduce reliance on technical expertise. Collaborative networks across academia, industry, and regulatory agencies will be essential to make these systems more broadly available and sustainable.

Finally, the path toward regulatory and ethical acceptance will strongly shape the future of OoC technologies. While regulatory agencies such as the FDA and EMA have begun acknowledging the value of OoC data in preclinical decision-making, formal validation frameworks are still in their infancy and rarely extend to veterinary applications. Ethically, OoC technologies align well with the 3R principles of replacing, reducing, and refining animal models. Their long-term impact, however, will depend on whether they can consistently deliver reproducible and clinically relevant data.

Looking ahead, the vision for OoC technologies is the development of multi-organ, patient-specific, and species-specific models that combine advanced bioengineering with real-time biosensing and computational analytics. In human medicine, this trajectory points toward personalized disease modeling and precision drug testing, while in veterinary applications, it promises to support precision livestock management, vaccine development, and comparative biomedical research. Realizing this vision will require sustained innovation, strong interdisciplinary collaboration, and the alignment of regulatory frameworks with emerging scientific capabilities.

## 7. Conclusions

The integration of organoids into microfluidic organ-on-a-chip (OoC) platforms is transforming both biomedical and veterinary research. These systems combine the physiological relevance of three-dimensional organoid cultures with the precision control of microtechnology, enabling the more accurate modeling of tissue–tissue interactions, disease processes, and drug responses. Advances in stem cell biology, biomaterials, and manufacturing technologies have already demonstrated the feasibility of reproducible, species-specific platforms for capturing complex physiological functions.

Despite these advances, significant challenges remain. Standardization in laboratories, the long-term stability of organoid cultures, and the scalable fabrication of chips remain limiting factors. Furthermore, the incomplete integration of vascular, immunological, and neuronal components limits the accuracy of current models. Solving these problems is crucial to ensuring that OoC systems evolve from proof-of-concept studies into robust, widely used tools.

OoC technologies will play a central role in precision medicine, drug discovery, and comparative veterinary applications. The development of multi-organ platforms, the integration of biosensors and artificial intelligence, and adaptation to regulatory frameworks will be crucial to maximize their translational impact. By bridging human and veterinary contexts, these systems not only support the replacement of and reduction in animal use in research but also open new avenues for One Health approaches that address common challenges across species.

## Figures and Tables

**Table 1:** A comparative summary of PDMS-based, thermoplastic-based, and hybrid OoCs.

	PDMS Chip	Thermoplastic Chip	Hybrid Hydrogel Chip
Primary Materials	PDMS	Thermoplastics	Hydrogel
Fabrication methods	– Soft lithography – 3D printing	– Hot embossing – Injection molding	– Micromolding – Channel definition – 3D printing/bioprinting – Laser ablation – Photolithography – Xurography
Merits	– Gas permeability – Optical transparency – Elasticity – Biocompatibility – Low cost	– Optical transparency – Mass production – Low absorption – Biocompatibility	– Biocompatibility – High permeability – Support 3D structure – Biodegradability
Limitations	– Absorption of small molecules – Difficulty in mass production	– Rigidity – Low gas – Permeability – High initial cost	– Low stiffness – Limited integrity
Experimental model	– Disease modeling – Study of mechanical stimuli	– Drug screening – Large-scale experimentations	– Soft tissue modeling – Vascular networks – Tissue barrier modeling
References	[62,63,64,65,66]	[69,70,72,73,74]	[77,78,79,80,81,82,83,84,85,86,87,88,89,90,91,92,93]

**Table 3:** The common methods used in OoC perfusion.

	Active Flow				Passive Flow
Pump	Syringe pump	Peristaltic pump	Pressure pump	Micropump	Gravity pump
Flow drives	motor-driven	compress tubing	gas pressure	electrical/ mechanical/ pneumatic actuation	gravity
Flow rate	µL/min-mL/min	µL/min-mL/min	nL/min-mL/min	nL/min-µL/min	low, variable
Flow accuracy	moderate	low	high	high	poor
Flow direction	unidirectional	recirculation	unidirectional	unidirectional	oscillatory
Flow stability	stable	less stable	very stable	stable	less stable
Tubing	bulky	bulky	bulky	-	-
Channels	single	multiple	multiple	single	single
Medium volume	small	large	large	small	medium
Application	– short- to medium-term studies – drug delivery	recirculating media	long-term perfusion	– portable – point-of-care microfluidics	– short-term diagnostics – simple perfusion organoid culture

## Data Availability

No new data were created or analyzed in this study. Data sharing is not applicable to this article.

## Abbreviations

The following abbreviations are used in this manuscript:

ADME	Absorption, Distribution, Metabolism, Excretion
AI	Artificial Intelligence
ASC	Adult Stem Cell
BMP	Bone Morphogenetic Protein
BMF	Boston Micro Fabrication
CFTR	Cystic Fibrosis Transmembrane Conductance Regulator
COC	Cyclo-Olefin Copolymer
COP	Cyclo-Olefin Polymer
CRISPR	Clustered Regularly Interspaced Short Palindromic Repeats
DDA	Data-Dependent Acquisition
DIA	Data-Independent Acquisition
DLP	Digital Light Processing
ECM	Extracellular Matrix
LQ	Label Quantification
LFQ	Label-Free Quantification
OoC	Organ-on-a-Chip
PC	Polycarbonate
PDMS	Polydimethylsiloxane
PDO	Patient-Derived Organoid
PEG	Polyethylene Glycol
PET	Polyethylene Terephthalate
PMMA	Polymethyl methacrylate
PS	Polystyrene
PSC	Pluripotent Stem Cell
PVA	Polyvinyl Alcohol
RGD	Arginine–Glycine–Aspartic Acid
SEBS	Styrene–Ethylene–Butylene–Styrene
SLA	Stereolithography
TEER	Transepithelial/Transendothelial Electrical Resistance
